# Use of numerical and spatial information in ordinal counting by zebrafish

**DOI:** 10.1038/s41598-019-54740-8

**Published:** 2019-12-04

**Authors:** Davide Potrich, Rosa Rugani, Valeria Anna Sovrano, Lucia Regolin, Giorgio Vallortigara

**Affiliations:** 1 Center for Mind/Brain Sciences, University of Trento, Rovereto, Italy; 2 Department of General Psychology, University of Padova, Padova, Italy; 3 Department of Psychology, University of Pennsylvania, Philadelphia, PA United States; 4 Department of Psychology and Cognitive Science, University of Trento, Rovereto, Italy

**Keywords:** Animal behaviour, Cognitive neuroscience

## Abstract

The use of non-symbolic numerical information is widespread throughout the animal kingdom, providing adaptive benefits in several ecological contexts. Here we provide the possible evidence of ordinal numerical skills in zebrafish (*Danio rerio*). Zebrafish were trained to identify the second exit in a series of five identically-spaced exits along a corridor. When at test the total length of the corridor (Exp. 1) or the distance between exits (Exp. 2) was changed, zebrafish appeared not to use the absolute spatial distance. However, zebrafish relied both on ordinal as well as spatial cues when the number of exits was increased (from 5 to 9) and the inter-exit distance was reduced (Exp. 3), suggesting that they also take into account relative spatial information. These results highlight that zebrafish may provide a useful model organism for the study of the genetic bases of non-symbolic numerical and spatial cognition, and of their interaction.

## Introduction

Extensive evidence support the existence of non-symbolic numerical skills in non-human species, which involve the extrapolation of numerical magnitudes from given sets of physical elements, without resorting to the use of abstract numerical symbols as adult humans do (reviews^1–5^). These skills have been documented in mammals^6–8^, birds^9–15^, amphibians^16,17^, reptiles^18^, fish^19–23^ and invertebrates^24–26^. A capability to process numerousness, known as “number sense”^27^, is helpful for individual survival in several important circumstances. In foraging situations, it enables the detection of larger food patches (mammals^28–31^; birds^32,33^; amphibians^34^). In social contexts, numerical judgements could be an effective anti-predatory strategy^35,36^: several fish species aggregate in big shoals of conspecifics in order to receive more protection against potential predators^19,21,22,37^. Moreover, a number sense helps to regulate intergroup conflicts in opponent rival groups^38–40^, the mating strategies in presence of potential competitors^41,42^ and parental care, with the aim to reduce brood parasitism^43^. Abstract and symbolic human mathematical abilities may therefore be rooted in an evolutionarily ancient and early developing number sense^2,4^.

Numerical competence studies have been focusing mainly on the proto-cardinal property of numbers, used in relative quantity judgements, proto-arithmetic operations and numerical comparisons. Nevertheless, another important aspect of numbers refers to “ordinality”. This refers to the information used to identify a position, or a rank, of a particular element in a series of identical ones (first, second, third…). Ordinality comprises also more advanced aspects, such as the capability to understand that, given a set of a certain number of elements, whenever an element is added, the magnitude of the resulting set is larger than the previous one and smaller than a subsequent one^44^. Ordinal competence has been extensively investigated in non-human species (e.g. primates^45–47^; birds^48,49^). Here, however, we should deal only with the most rudimentary aspect of ordinality, namely the ability to identify a stimulus on the basis of its position in a series of identical stimuli.

Rats proved to be capable of learning to select and enter a target tunnel on the basis of its ordinal position in an array of six. The task was learned relatively rapidly and the performance was maintained after alteration in size and shape of the array of tunnels^50,51^. Similarly, Rugani and colleagues^52^ showed that chicks were able to identify the 3^rd^, the 4^th^ or the 6^th^ element in a series of 10 identical elements. Moreover, when tested with a new series in which the inter-elements distance was changed, chicks relied on the ordinal numerical position rather than on the absolute distances. Pigeons^10^ and honeybees^53,54^ showed similar abilities.

The use of serial information has been documented also in a fish species, the guppy (*Poecilia reticulata*)^55^. Guppies learned to identify a feeder (i.e., the 3^rd^ feeder) in a fixed series of identical ones (i.e., 8 feeders) even when the feeder position and the distance between feeders were varied from trial to trial, preventing them from using spatial strategies. In a further experiment, to assess which strategy guppies spontaneously use when both ordinal and spatial cues are simultaneously available, fish were trained with a fixed feeder position and inter-feeder distance. When, in probe trials, the two sources of information were placed in conflict, fish spontaneously used ordinal rather than spatial cues. Moreover, guppies showed a decrease of precision as the ordinal position of the target increased, making more errors in selecting for example the 5^th^ position (in a series of 12 feeders) rather than the 3^rd^ one.

**Figure 1:**
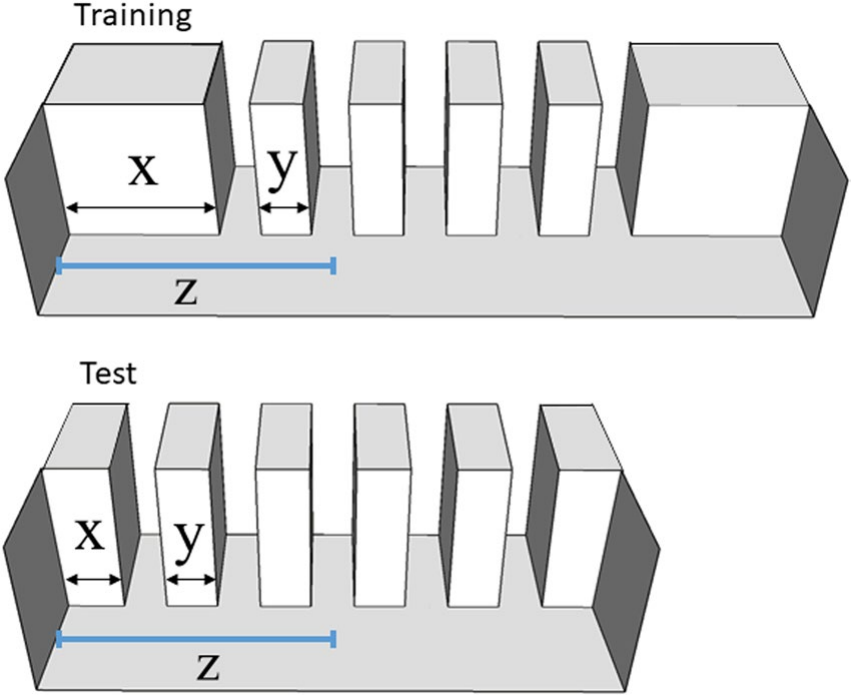
Schematic representation of the corridor used in Experiment 1. At training, fish were trained to select the second exit (from the left side, in a frontal view). At test, the length of the extremities of the corridor (x) decreased, while the inter-exit distances (y) did not change. As a result, the total length of the series of exits remained fixed, but the total corridor length was reduced creating a conflict between ordinal and spatial information. During testing, the 3^rd^ exit was in the position previously occupied at training by the target one (the 2^nd^), as indicated by the blue lines (z).

**Figure 2:**
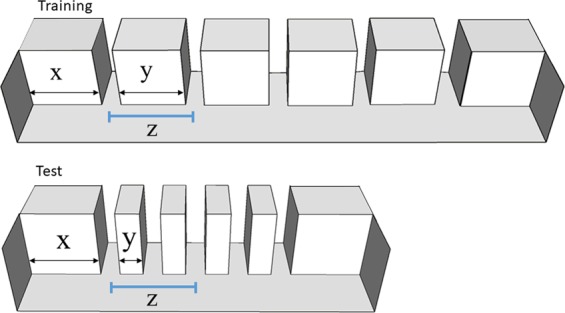
Schematic representation of the corridor used in Experiment 2: during training and test, the length of the corridor extremities (x) did not change, while the inter-exit distances (y) were reduced. In this way, the total length of the exits’ series changed, as well as the total corridor length. In the testing series, the 3^rd^ exit was in the position previously occupied by the second one, as indicated by the blue lines (z).

The aim of this study was to investigate whether zebrafish could afford encoding of ordinal numerical information. Apart from extending to another species comparative evidence for ordinality cognition, the research was motivated by the possibility and prospect associated with the use of an animal model that in recent years has become an established reference model for the studies of the genetic bases of behaviour (reviews^56–60^). Here we carried out three experiments. In Experiment 1, zebrafish were trained to identify a target exit (i.e., the second one) among a series of five identical exits, equally spaced along a corridor. During training, both spatial and ordinal information were available, preventing us from understanding which strategy was spontaneously used. To disentangle this, at test, the total length of the corridor was reduced (decreasing the distance between the corridor extremities), while the total length of the exits’ series, as well as the inter-exit distance, were identical, so that the third exit was located at the spatial position previously occupied by the target (Fig. 1). As a result, the total length of the series of exits was fixed, but the total corridor length was reduced, creating a conflict between ordinal and spatial information. Fish showed a preference for the numerical correct exit. However, since the inter-exit distances were identical in training and test, zebrafish could have relied on the distance between the target exit and the beginning of the series or, in some instances, the exit directly to the left of the release site. We checked for this in Experiment 2, in which the inter-exit distance was reduced from training to test, so that at test the third exit was located in the spatial position previously occupied by the target (the second; Fig. 2).

**Figure 3:**
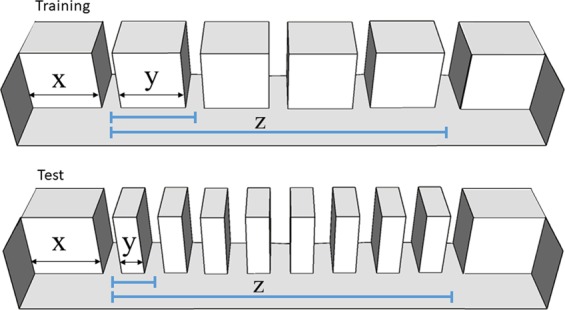
Schematic representation of the corridor in Experiment 3: during training and test, the length of the corridor extremities (x) did not change, while the inter-exit distances (y) were reduced and the number of exits was increased (from 5 to 9). The total length of the exits’ series and the entire corridor length did not change. The blue lines (z) show how the relative proportion between the inter-exit distance and the entire exits’ set is not maintained between training and test.

These experiments showed that zebrafish did not take into account absolute spatial distances; they instead selected the target exit that referred to the ordinal position. This evidence still did not exclude the possible use of relative spatial information. In Experiment 3, we wanted to understand whether fish were using the relative spatial distance between the target exit with respect to the entire length of the exits’ series. We checked for this hypothesis by retraining fish in the same apparatus as in Experiment 2 (target still in the second exit). At test we (i) reduced the inter-element distance and (ii) increased the number of exits (Fig. 3). In this case, a choice for the third exit would suggest a use of relative spatial distance; in contrast, a preference for the second exit would prove the use of ordinal information. Overall our findings suggest that zebrafish may rely on both ordinal and relative spatial cues.

## Results

**Figure 4:**
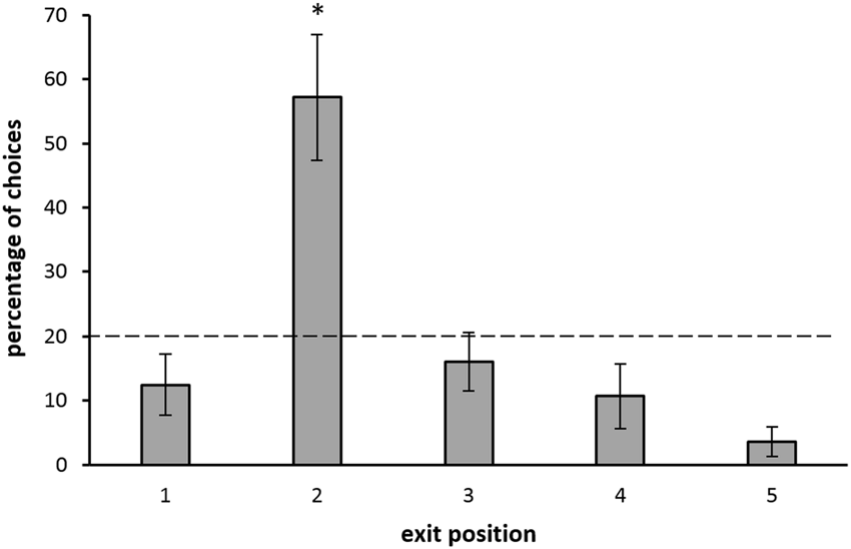
Experiment 1: Percentages of first choices (mean ± SEM) for each exit at test (sample size = 7). The dotted line (y = 20%) represents chance level. Asterisks (*) indicate a significant difference from chance (*p* < 0.05).

In Experiment 1, 7 zebrafish learnt to identify the target exit in a series of five identical ones in the training phase.The mean number of trials needed to reach the learning criterion was 176.29 ± 27.74 (mean ± SEM). Among the five exits, only the target one (the second) was selected above chance level (t(6) = 8.255, *p < *0.001). The second exit was chosen significantly more, even when compared to the two adjacent exits (second *vs*. first: t(6) = 5.546, *p = *0.001; second *vs*. third: t(6) = 5.348, *p = *0.002). At test we scored only the first choices (i.e., the first selected exit) and we computed the percentage of choices for each exit in a total of 8 test trials (Fig. 4). At test, the change in the corridor length did not affect the zebrafish performance in spite of the potential conflict between ordinal and spatial information (in fact, at test, the third exit was in the position occupied during training by the second one that was the target). A Friedman test revealed a statistically-significant difference in the fish’s responses among the five exits (χ^2^(4) = 12.419, *p* = 0.0145); only the second one was selected above chance level (One-sample Wilcoxon Test: V = 27, *p* = 0.017). The second exit was selected significantly more often than the third one (Related-Sample Wilcoxon Test: W = 26.5, *p* = 0.041) and the first one (W = 27.0, *p = *0.034).

**Figure 5:**
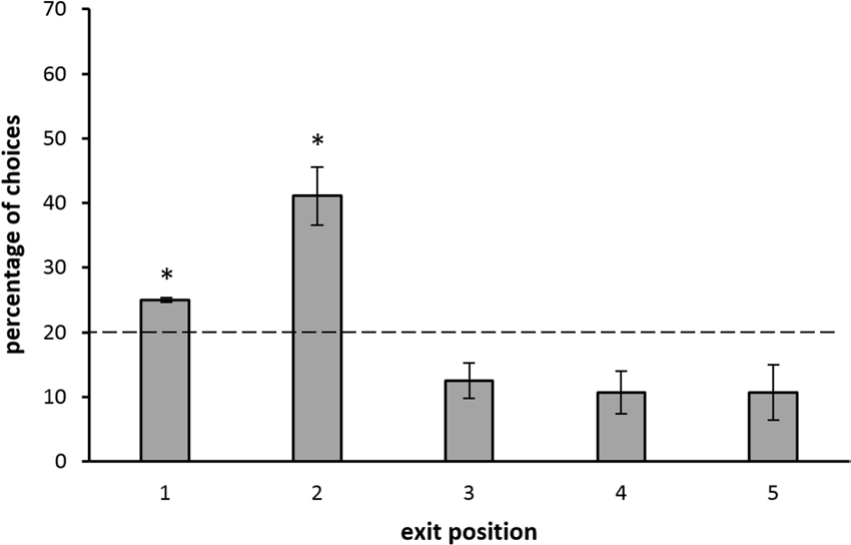
Experiment 2: Percentages of first choices (mean ± SEM) for each exit at test (sample size = 7). The dotted line (y = 20%) represents chance level. Asterisks (*) indicate a significant difference from chance (*p* < 0.05).

In Experiment 2, at training, 7 new fish reached the learning criterion in 108.43 ± 18.10 (mean ± SEM) trials. The target exit (the second) was selected well above chance level (t(6) = 10.293, *p < *0.001). The second one was also chosen significantly more often than the adiacent ones (second *vs*. first: t(6) = 5.054, *p = *0.002; second *vs*. third: t(6) = 6.314, *p* < 0.001). No significant difference between Experiment 1 and Experiment 2 in the number of trials needed to reach the learning criterion was found (t(12) = 2.048, *p* = 0.063). Test results are shown in Fig. 5. A Friedman test revealed a statistically significant difference among the five exits (χ^2^(4) = 20.623, *p* = 0.0038). The second exit was chosen above chance level (V = 28.0, *p* = 0.020) as well as was the first exit (V = 28, *p* = 0.015). Moreover, the second exit was selected significantly more often than the first one (W = 28.00, *p* = 0.031) and the third one (W = 28.0, *p* = 0.021). The prefence for the first exit (which is not related to ordinal or spatial information) is however easily explained by the tendency of fish to direct their choices towards the most salient elements that were at the extremities of the series. Similar behaviors have been already described in other animal species whenever a similar paradigm was used (chicks^14^; rats^50^).

**Figure 6:**
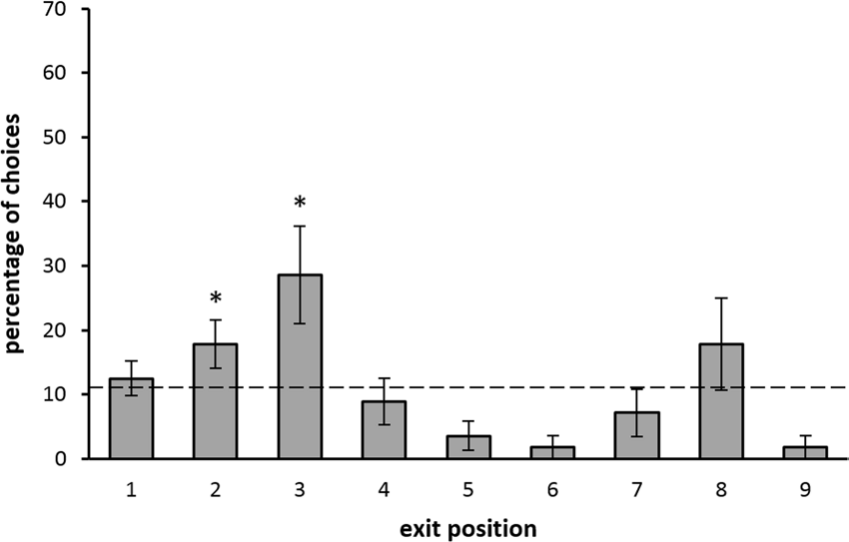
Experiment 3: Percentages of first choices (mean ± SEM) for each exit at test (sample size = 7). The dotted line (y = 11.11%) represents chance level. Asterisks (*) indicate a significant difference from chance (*p* < 0.05).

In Experiment 3, the same group of fish that performed Experiment 2, after a retraining phase, was tested. At test, a statistically significant difference among the nine exits emerged (χ^2^(8) = 20.743, *p* = 0.00786; Fig. 6). When the choices emitted for the numerically correct exit (the second) and the spatially correct exit (the third) were compared with chance level (one-tailed test), a significant difference for both exits emerged (respectively: V = 25.00, *p* = 0.035 and V = 25.00, *p* = 0.037). No significant difference was found when we compared the performance on the second and the third exit (W = 5.00, *p* = 0.281). Noteworthy, fish showed a trend preference for the eighth exit (which corresponded specularly to the second exit from the right corridor side); this was not observed in the two previous experiments, although this preference was not above chance level (V = 19.00, *p = *0.221) and not significantly different from the seventh exit (W = 5.00, *p = *0.423). Similarly to Experiment 2, fish showed a choice trend for the first exit; although it was not above chance level (V = 22.00, p = 0.094), this suggests that the first exit is a particular salient position for the animals since it is at the beginning of the exits’ series.

Considering that the subjects were always confined into the starting area at the center of the corridor, an alternative explanation of the results might be that fish identified the target exit (the second) simply using a direct path from the starting point (i.e., the correct exit was the central exit to the left), without relying on any ordinal information. In Experiment 1, we observed that, among all the 56 trials (8 test trials for 7 subjects), a direct approach to any corridor occurred in 15 trials, 5 of which were directed towards the second exit (Binomial Test, *p* = 0.21). This suggested that fish did not use the distance from the starting point as a strategy to identify the target exit (second one). Similarly, in the two other experiments, zebrafish showed a direct approach from the starting point only in 1 trial out of 56 in Experiment 2, and in 8 out of 56 trials in Experiment 3 (one of which was directed to the second and one to the third exit).

Moreover, the possible use of the central exit as a landmark to identify the target exit was considered. In all the test trials we computed whether the selected exits were approached from either the right side (i.e., swimming from the apparatus’ central exit) or from the left side (i.e., swimming from the left apparatus’ end). We observed that, in Experiment 1, the second exit has been approached for 53% from the right side. In Experiment 2, the first and the second exits have been respectively approached from the right side for 20% and for 23% of the time. Lastly, in Experiment 3, the second exit has been selected from the right side 50%, whereas the third one 35% of the time. These data clearly do not support the hypothesis that fish may have used the central exit as a cue to identify the target exit.

## Discussion

Overall, the results of the experiments suggest that zebrafish may use a mixture of purely ordinal (numerical) information and relational distances to localize the target exit.

When spatial cues conflicted with numerical ordering (Exps. 1 and 2), zebrafish seemed not to rely on the absolute distances, choosing the exit on the basis of its numerical order and/or the relative spatial information. When relative distances (i.e., distance of the exit with respect to the overall length of exits’ series) were in conflict with numerical ordering (Exp.3), zebrafish showed a mixed performance, choosing both alternatives, the spatial and the numerical ones. These results in zebrafish do not fully agree with evidence in guppies^55^ that seemed completely unaffected by spatial information, either absolute or relative. Similarly, evidence in mammals^50,51^, birds^52^ and insects^54^ showed an advantage of ordinal over spatial cues.

The different performance between zebrafish and guppies could be related to differences between the species (*Danio rerio* belongs to the Cyprinidae family and *Poecilia reticuata* belongs to the Poeciliidae) that live in different ecological niches. Moreover, the different setup used here could represent an additonal factor, since the set of exits was located along a narrow corridor that could have led to the use of spatial information in concurrence with the ordinal one.

In Experiment 1, we controlled for the possible use of spatial cues provided by the distance between the end of the corridor (the lateral edge) and the target exit. To this aim, at test we modified the corridor length by reducing the distance between the two edges. Results showed that zebrafish do not rely on this distance. However, since the inter-exit distances were identical during training and test (i.e. “y” distance in Fig. 1), zebrafish could have relied on the distance between the target exit and the beginning of the exits’ series. This possibility was tested in Experiment 2.

In Experiment 2, when at test a conflict between ordinal and absolute spatial information occurred, zebrafish relied more on the ordinal one. However, the high preference for the second exit at test could be also related to the use of relative spatial information and not only to pure numerical information: at training, fish could have learnt to select the target exit (the second) in relation to its spatial position among the others. At test, although the corridor and the inter-exit distance were reduced, the spatial proportion among the elements was mantained. To disentangle this last ordinal-spatial concurrence, zebrafish performed a further test (Experiment 3) in which the relative spatial distances and the ordinal position were in conflict with each other, revealing that both strategies were used.

In Experiment 3, the use of spatial and ordinal cues might be related to the high number of changes, which occurred between training and test (i.e., the change of relative spatial distances and the number of exits). In a natural enviroment, serial ordinal information may be in some cases more reliable than other types of information such as shapes, colors or texture: rocks, trees and inlets provide fixed elements that rarely change position among time and seasons. Nevertheless, we found that in zebrafish the relative position of the elements is as important as the ordinal rank information; a strong variation of the elements’ series (such as the number of the exits) may picture a really different environment, leading the fish to use both spatial and ordinal (numerical) information to maximize their success in localizing the target. These findings are consistent with other studies showing that in numerical tasks, as the task become more difficult, a redundancy of information is needed to solve it^61,62^.

Interestingly, in Experiment 3, a trend for selecting the eighth exit, which specularly corresponded to the second exit from the right side, was present. Although not statistically significant, the change of relative spatial distances between training and test, combined with an increase of exits available, could have led the fish to start exploring the series even from the right side. Moreover, no trend preference for the seventh exit was found, (which is supposed to be the specular exit to the third one from the right side referring to spatial cue); this may suggest that exploration from the right side prompt for the use of ordinal information rather than of the spatial one.

In Experiment 2, besides the strong preference for the second exit (ordinal cue) over the third one (spatial cue), zebrafish chose the first exit at a level above chance. This effect is likely to be associated to perceptual salience of the external elements. A similar trend in approaching the first exit, even if less strong, appeared also in Experiment 3. In all the experiments fish learnt to select a target exit in a series of identical exits. They appeared to firstly learn to identify the “beginning of the series” (i.e. the left end), relying on their relative position in the apparatus: the exits’ series was on their right side when they faced the first exit on the left and on their left side when they faced the opposite direction. This is a well proved ability that fish use in navigating and in orienting in their environment^63,64^. Here it appears that fish used this kind of information to identify the beginning of the series. This also explains why fish made more errors on the first left exit and not on the first right one. Similar phenomena of attractivity on initial and terminal elements in a series have been described in similar tasks in other species, such as rats^50^ and domestic chicks^14^.

In recent years zebrafish have become established as a developmental and behavioral genetic model species. Their popularity has arisen from the relative ease with which one can generate mutant and transgenic lines, and the transparent nature of embryonic and larval forms that facilitates cell biological analysis of identified phenotypes. Zebrafish display a range of behaviors of translational relevance to human cognition^60,65^. Nevertheless, there is a lack of behavioral techniques that investigate such cognitive abilities in this animal model. In fact, most of the developed behavioral tasks investigate simple behavioral activity (e.g. behavior in early life: escape response, phototaxis, hunting; social behaviors: shoaling, aggression and mating; for a review see^66^). With this study we provided evidence for numerical (ordinal) and spatial skills, which could be of broad relevance to a range of biological disciplines. It has been hypothesized that non-symbolic numerical abilities are based on an evolutionary conserved system, whose impairment is thought to underlie human dyscalculia. This is a clinical syndrome in which a specific domain, involving numerical abilities, is compromised in absence of a general cognitive deficit^67,68^. The use of zebrafish as a behavioral model could provide a link between the investigation of the underlying molecular bases of numerical cognition and the genetic mechanisms of dyscalculia.

## Method

### Subjects and rearing conditions

We used 14 adult males of zebrafish, *Danio rerio* (fish size ranged between 4 and 5 cm in length; average age: 6 month-old). A group of fish (N = 7) took part at Experiment 1. A second group (N = 7) took part in both Experiment 2 and Experiment 3. The sample size of N = 7 subjects, which is the same for all the experiments and consistent with the one of our previous works^22^, has been estimated a priori conducting a power analysis with the program G*Power 3.1.9.4^69^. In this analysis we used the average effect size obtained in the different conditions of a previous study^22^, that investigated numerical abilities in the same (average of Exp. 1 and Exp. 2). The power analysis for the Wilcoxon signed-rank test revealed that a sample of 7 subjects was required to detect a significant effect with power (1 - β) set at 0.80, effect size (*d*) = 1.489 and alpha = 0.05, two tailed. Moreover, the choice of the sample size is in accordance with the ethical method of “Reduction” in favor of which, the least possible number of animals should be used in obtaining consistent results (see also “Ethical Regulations”).

Fish were purchased from local commercial suppliers and maintained in our laboratory in an automated aquarium system (ZebTEC Benchtop, Tecniplast) in 3.5-litre plastic tanks. Because the sexual dimorphism is low in this species, to ensure that we tested only males and used females only as social and sexual attractive stimuli, fish were housed separately in groups of males and females of 10 individuals each. Water temperature was maintained at 26 °C and the system was illuminated under a 12:12 light/dark cycle. Fish were fed three times a day with dry food (Sera GVG-Mix). Once each fish was selected to perform the experiment for the first time (randomly chosen from the housing tank), it was moved into a 20-litre glass aquarium (23 × 38 × 25 cm) set with gravel and vegetation, mimicking a natural environment. The aquarium was partitioned in different areas each housing a single fish. Each area was separated one from another by a transparent net divider, allowing the fish to see each other (preventing isolation effects) but remaining also recognizabe to us. One at a time, each fish was singly picked up from its own partition to perform the experimental session and placed back at the end of it. During the entire experiment duration, fish that were not performing the task (training or test session), were kept separated in their own acquarium partition and were fed only during the experimental sessions (inside the experimental apparatus). In all the experiments, adult females of zebrafish were used as social and sexual reward for the male subjects^70^.

## Experiment 1

### Apparatus

**Figure 7:**
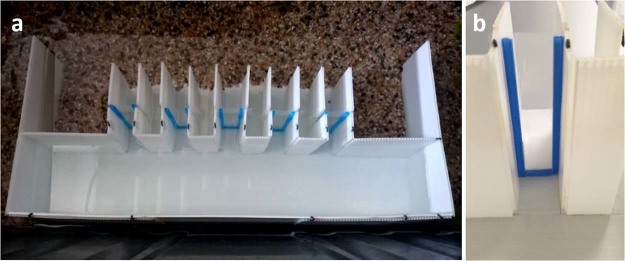
(**a**) A photograph of the corridor used in Experiment 1. (**b**) Detail of an exit.

The experimental setup, used during the training phase, was a large black arena (110 × 45 × 52 cm). This was divided in two connected sites: the corridor and the reinforcing area. The corridor was a white plastic rectangular tank (50 × 9 × 11 cm). Along one of the two long walls of the corridor there was a series of 5 exits (each exit was 9 cm length, 3 cm width, 11 cm height; see Fig. 7a). The series was centred on the corridor longer wall and its beginning was placed at 10.5 cm from each wall extremity. During the training phase, the inter-exit distance was 3.5 cm and the total length of the series was 29 cm. Each exit was marked by a blue plastic frame (5 mm thick) located at 3.5 cm from the exit entrance (Fig. 7b). All the exits, except the target one, in correspondence of the blue frame had a plastic transparent sheet, that did not allow the fish to leave the corridor. As a result, all the exits looked the same, but only the second one allowed the fish to leave the corridor and enter in the reinforcing area. This area was attractive for fish, because it was set with gravel, plants and a fish net breeder (16 × 12 × 12 cm) containg two female conspecifics, which were released whenever the experimental subject gave a correct response.

During the test phase, the corridor was identical except for the length of the longest walls. In the new corridor, the distance between the beginning of the series and the extremities of the corridor was reduced to 4 cm, while, the inter-exit distance (3.5 cm) as well as the number of exits (5) did not change from training to test. Therefore, the total length of the corridor was 37 cm (instead of 50 cm). As a result, considering the whole generalization corridor (see Fig. 1), the distance between the beginning of the corridor and the 3^rd^ exit was now at the same absolute distance where the 2^nd^ exit (the target) was in the training corridor. However, considering only the whole series of exits, the distance between the beginning of the series and the 2^nd^ exit was identical during training and test. To avoid any differential reinforcement, during test all the exits were blocked (extinction procedure).

The water in the experimental apparatus was maintained at a constant temperature of 26 °C and kept clean by a pump and a filter system (Micro Jet Filter MCF 40). The apparatus was lit by a fluorescent 18-W neon lamp, and a webcam (Microsoft LifeCam Studio) placed above the setup (50 cm) recorded fish behavior.

### Procedure

The experimental procedure was divided into two parts: a *training* phase and a *test* phase.

### Training

During the training phase fish could leave the corridor only via the target exit, since the others were blocked. From a frontal view, the five exits looked identical but the only one which allowed the fish to leave the apparatus (i.e., the target one) was always the second one from the left end.

Fish daily underwent an experimental session. This consisted of 10 valid trials for each fish, that occurred at the same time every day. Testing time for all fish ranged from 9 am to 3 pm. Before each session, the fish was taken from the housing aquarium and left for 5 minutes in the reinforcing area with the females conspecifics. At the beginning of each trial, the fish was gently taken, using an aquarium fish net, and confined for 15 seconds into a transparent plastic cylinder (6.5 cm in diameter and 12 cm in height) located in the center of the corridor. The fish was then released, allowing it to freely move within the corridor. For each trial, the animal’s behavior was observed, recording the number of attempts to enter each exit, until the target one was identified. It is important to point out that, although the starting position of the fish was always in the center of the apparatus, zebrafish did not show the use of a “path” strategy, swimming directly toward an exit. They rather explored the environment swimming along the corridor several times before making a choice; usually they did arrive close to the edge without touching it. Given the position of the fish and the wide visual field of this species, the corridor edge, as well as the exits’ series are clearly visible from fish’s perspective in the recorded turning point (see video example in the electronic Supplementary Material and results section). A choice was considered done when the fish entered the exit, at least with ¾ of its body. Each trial could last up to 15 minutes and was considered valid if the animal chose at least one exit. If the fish did not choose any exit in this time, the trial was considered null and it was repeated in order to obtain, at the end of the session, a total of 10 valid trials. After two consecutive null trials the daily experimental session ended. The reward obtained at the end of each trial depended on the animal’s performance using a correction method^71^. When the target exit was chosen as first attempt (i.e., no other exits were selected before the target one), the fish received a small quantity of food and was allowed to freely swim in the reinforcing area and interact with the two female conspecifics, which were released from the net breeder, for 6 minutes. When the animal first visited other exits and then entered the target one, it was allowed to spend 3 minutes in the reinforcing area, moreover it did not receive any food reward and it was not allowed to interact with the females, which remained confined in the net breeder. This standard correction method and the possibility to enter in an external enriched region as reinforcement have been used in previous works with fish (e.g. refs. ^72–74^), revealing that it is optimal in tasks involving an operant conditioning procedure. At the end of each daily session, the number of choices for each exit was calculated, and the number of correct choices was compared with the number of choices for each other exit. Learning was considered achieved when the target was selected, in two consecutive daily sessions, at least 60% more than each other exit and at least 40% of the total choices for all five exits. These learning criterions have been adopted because we wanted fish to select the target exit above the chance level (i.e., 20%) and more than each other exit.

### Test

The test took place one day after the learning criterion was reached. A complete test comprised 8 trials, divided in two sessions of 4 trials, which occurred in two consecutive days. Before each testing session, the fish underwent a series of rewarded recall trials (exactly as in the same training procedure) to reinstate the motivation of the animals immediately before the test phase. As soon as the fish emitted two consecutive correct choices (this required between 2 and 6 trials) it underwent the testing phase. If the subject did not satisfy this criterion, it underwent a complete daily training session of 10 trials and the test was repeated the next day. To avoid any learning during testing, all the exits were blocked and the test was conducted in extinction procedure. At the beginning of each test trial, the fish was placed in the middle of the corrridor, into the transparent cylinder for 15 seconds. It was then released and allowed to perform its exit attempt for a maximum time of 10 minutes. If the fish did not choose any exit in this time, the trial was considered null and it was repeated in order to have at the end 8 valid trials. For each testing trial, only the first choice was considered. At the end of each trial, the fish was relocated into the outer reinforcing area (the same as in the training phase), leaving it free to swim for 5 minutes (inter-tests interval). No food was provided during the entire test phase.

## Experiment 2

Fish were trained to identify the second exit from the left (target). The corridor used during training was identical to the one used in Experiment 1, with the only exception being its dimensions. Here the longest walls measured 76 cm, and the inter-exit distance was 10 cm. The distance between the corridor extremities and the beginning of the exits’ series was 10.5 cm.

### Procedure

Fish were trained to choose the second exit from the left side, as in Experiment 1. At test, the inter-exit distance was manipulated, decreasing from 10 cm to 3.5 cm (corridor total length at test was 50 cm, see Fig. 2). These changes created a conflict between ordinal position and spatial distance. Doing so, the distance between the first and the second exit at training was the same as the distance between the first and the third exit at test. This condition allowed to further understand whether the strategy used by zebrafish at training was based on spatial distances or on ordinal information.

## Experiment 3

In this experiment the same group of *Danio rerio* which had undergone the previous experiment has been used. At the end of the second experiment, fish underwent a retraining phase, in the same apparatus in which they had been previously triained with the same total length of the corridor (76 cm) and inter-exit distance (10 cm) as in Experiment 2 (the procedure and the learning criterion is the one described for Experiment 1). During testing, the number of exits changed from 5 to 9 (Fig. 3) and the inter-exit distance was reduced from 10 to 3.5 cm. In this way, the number of exits increased, albeit the total length of the series remained identical. This allowed evaluating whether these relative distances mismatch affect the fish’s performance.

### Data analysis

All the data have been analyzed by using “JASP” and “SPSS” statistics programs. At the end of the training session, the percentage of the frequencies of choices for each exit was compared with chance level (One-Sample *t* test) and between exits (Paired-Sample *t* test). At test, the results were analyzed taking into account the percentage of first choices for each exit. A non-parametric statistical test was used to analyze and detect differences among the exits (Friedman Test). We used One-Sample Test and Related-Sample Tests (Wilcoxon signed-rank) to analyze departures from chance level. An inter-observer reliability criterion was applied in the re-coding of a subset of 10% of different videos (*p* < 0.001, Pearson’s correlation between the ratio calculated on the original coding and on the *de novo* coding performed by an experimenter blind on the test condition of the fish).

## Ethical Regulations

The present research was carried out at the Animal Cognition and Neuroscience Laboratory (ACN Lab) of the CIMeC (Center for Mind/Brain Sciences), at the University of Trento (Italy). All husbandry and experimental procedures complied with European Legislation for the Protection of Animals used for Scientific Purposes (Directive 2010/63/EU) and were previously authorized by the University of Trento’s Ethics Committee for the Experiments on Living Organisms, and by the Italian Ministry of Health (auth. num. 1111/15-PR, prot. num. 13/2015 and auth. num. 893/2018-PR). The number of animals used in the experiments is closely consistent with the alternative method of “Reduction”, using only the minimum number of animals useful to draw statistically valid and powerful enough conclusions.

## Supplementary information

Dataset 1

Training trial video example

## Data Availability

Data are available in a submitted Supplementary file.
